# Cytogenetic and Molecular Characterization of *Sphaerophoria rueppellii* (Diptera, Syrphidae)

**DOI:** 10.3390/insects16060604

**Published:** 2025-06-08

**Authors:** Pedro Lorite, José M. Rico-Porras, Teresa Palomeque, Mª Ángeles Marcos-García, Diogo C. Cabral-de-Mello, Pablo Mora

**Affiliations:** 1 Department of Experimental Biology, Genetics Area, University of Jaén, Paraje Las Lagunillas s/n, 23071 Jaén, Jaén, Spain; jmrico@ujaen.es (J.M.R.-P.); tpalome@ujaen.es (T.P.); 2 Unidad Asociada de I+D+i al CSIC “Interrelaciones Insecto-Patógeno-Planta y Sus Agentes de Biocontrol” (IPAB), Research Institute CIBIO (Centro Iberoamericano de la Biodiversidad), Scientific Park, University of Alicante, Ctra. San Vicente Del Raspeig s/n, 03690 San Vicente del Raspeig, Alicante, Spain; marcos@ua.es; 3 Department of General and Applied Biology, Institute of Biosciences/IB, UNESP—São Paulo State University, Rio Claro 13506-900, São Paulo, Brazil; cabral.mello@unesp.br

**Keywords:** *Sphaerophoria rueppellii*, Syrphidae, mitochondrial genome, cytogenetics, karyotype, phylogeny

## Abstract

*Sphaerophoria rueppellii* is a Palearctic hoverfly widely used as a native biocontrol agent against aphid pests in Mediterranean agroecosystems. In this study, we present a cytogenetic analysis and characterization of the mitochondrial genome of this species. Chromosomal preparations, obtained from third-instar larvae, were used for conventional staining, DAPI staining and C-banding techniques, and major ribosomal DNA (rDNA) location by fluorescence in situ hybridization (FISH). Karyotype analysis revealed a diploid number of 2n = 10, with heterochromatic blocks in the pericentromeric regions of all autosomes and rDNA clusters on both sex chromosomes. The complete mitochondrial genome (16,605 bp) was sequenced and annotated using next-generation sequencing and assembly pipelines. It contains the typical 37 mitochondrial genes and a highly A + T-rich control region with tandem repeats. Gene order and codon usage were conserved compared with other Syrphidae. Phylogenetic reconstruction based on mitochondrial protein-coding genes clarifies the species’ placement within the Syrphini tribe. Our results contribute valuable genomic and cytogenetic information that supports comparative analyses and may aid in taxonomic clarification within the genus. These findings also offer key data that could guide the genetic optimization of *S. rueppellii* as an efficient, environmentally safe biological control agent in sustainable agriculture.

## Simple Summary

Hoverflies or flower flies (Syrphidae) are a diverse group of dipterans, with more than 6600 species worldwide. This insect group has high relevance to pollination and the regulation of insect pest populations. *Sphaerophoria rueppellii* is a hoverfly widely distributed across the Palearctic region, particularly common in Mediterranean ecosystems and crops. It is already used commercially to control aphid pests and, due to its strong trophic preference for aphids, it is effective without damaging agroecosystems. Because it is native to these areas, its use in biological control avoids the risks associated with introducing non-native species. This study provides new genetic and cytogenetic information on *S. rueppellii*, including its chromosomes and mitochondrial genome, to help improve the understanding of its evolutionary relationships.

## 1. Introduction

Syrphids (Diptera, Syrphidae), also known as hoverflies or flower flies, are a family with 6674 species and 284 genera distributed worldwide [1], making them one of the largest dipteran families. This diverse family of flies exhibits a striking mimicry to hymenopterans such as bees and wasps, with which they are often mistaken. Adults are frequently found on flowers, feeding on nectar and pollen [2], and in some cases, they can also act as effective pollinators [1,3]. The larvae of syrphids have a very different and highly varied biology compared to the adults, displaying diverse feeding habits. Syrphids are currently classified into four subfamilies [4,5] based on integrated taxonomy (larval and adult morphology, molecular data, and larval feeding habits). The larvae of Eristalinae species are phytophagous and saprophagous, while those of Microdontinae, Pipizinae, and Shyrphinae are predatory. Predatory species mainly feed on soft-bodied Hemiptera species, such as aphids (Aphidoidea) and psyllids (Psylloidea) [6], which makes some of them valuable for biological pest control [7,8].

*Sphaerophoria rueppellii* Wiedemann, 1820 is widely distributed throughout the Palearctic region, being particularly abundant in Mediterranean natural ecosystems and crops. Because *S. rueppellii* is native to the Mediterranean region where it is applied, its use in biological control avoids the ecological risks typically associated with the introduction of non-native species, such as invasive behavior, the disruption of trophic interactions, or the competitive displacement of endemic fauna. The environmental conditions within its range allow for optimal predatory activity against aphid pests without causing harm to the biocenosis (as seen with invasive species) or leading to ecological imbalances (such as displacement or competition). Currently, this species is commercially available as a biological pest control agent [2,9,10]. The species has also been the focus of detailed studies on larval morphology and aspects of its reproductive biology, highlighting its importance as a well-studied and ecologically significant predator [11,12,13].

Cytogenetic studies in insects are essential for understanding their genetic diversity, evolutionary relationships, and population dynamics. They provide insights into karyotype evolution, sex determination mechanisms, chromosomal rearrangements, and their impact on species diversification [14]. Chromosomal rearrangements play a significant role in shaping adaptability and resistance to environmental changes, as they can lead to genetic differentiation, reproductive isolation, and even speciation, as observed in model organisms such as *Drosophila pseudoobscura* Frolova, 1929 and *D. persimilis* Dobzhansky and Epling, 1944 [15]. In applied entomology, the study of chromosomal and genomic structures has been fundamental to improving pest control strategies. Cytogenetic research conducted over the past three decades on fruit flies of the family Tephritidae has significantly supported the development of sterile insect techniques (SITs) [16]. More recent genomic analyses, such as those performed on *Ceratitis capitata* Wiedemann, 1824, have identified temperature-sensitive lethal regions that are useful for improving genetic sexing strains and enhancing the efficiency of SIT programs [17]. Additionally, understanding karyotypic diversity in beneficial insects such as butterflies, parasitoids, and pollinators provides critical insights into their evolutionary potential and ecological adaptability. A remarkable example is found in the butterfly genus *Agrodiaetus*, whose species exhibit extraordinary variability in chromosome numbers. This karyotypic diversity has been linked to rapid and effective speciation mechanisms, as chromosomal rearrangements may act as reproductive barriers that promote differentiation among closely related populations [18]. Collectively, these findings contribute to the optimization of beneficial insect use in sustainable pest management and biodiversity conservation programs.

The analysis of mitochondrial DNA sequences in insect species is essential for phylogenetic studies, molecular identification, and conservation [19,20,21]. As mitochondrial DNA is maternally inherited and exhibits a relatively high mutation rate, it enables the differentiation of cryptic species and facilitates a more precise assessment of evolutionary relationships [22]. Additionally, studying mitochondrial DNA helps to elucidate genetic variability and ecological adaptation, key factors for improving the effectiveness of insects in biological pest control [23]. Moreover, it provides valuable insights into the demographic history of populations and their resilience to environmental changes [24,25,26].

Despite its relevance as a biological control agent in Mediterranean agroecosystems, detailed genetic information on *S. rueppellii* remains scarce. Previous studies have provided only basic insights into its karyotype, without thorough analysis of its heterochromatin distribution or ribosomal gene organization. Similarly, its mitochondrial genome has not yet been fully characterized or systematically compared with related species. These knowledge gaps limit our understanding of its genetic diversity and evolutionary placement within Syrphidae. This study aims to provide a cytogenetic and molecular characterization of *S. rueppellii.* The objectives include analyzing its karyotype and heterochromatin distribution, determining the location of its rDNA genes, and sequencing and annotating its complete mitochondrial genome to explore gene organization, codon usage, and structural features. Additionally, the study conducts phylogenetic analyses to clarify the placement of *S. rueppellii* within the Syrphinae subfamily. By generating foundational genetic and cytogenetic data, this research supports the evaluation of genetic diversity, evolutionary stability, and taxonomic consistency within the genus. These insights are essential for developing targeted breeding and conservation strategies, improving mass-rearing efficiency, and ensuring the safe and effective application of *S. rueppellii* as a native, environmentally sustainable biological control agent.

## 2. Materials and Methods

### 2.1. Material and Chromosome Preparation

Larvae and pupae of *Sphaerophoria rueppellii* (Syrphidae, Syrphinae, Syrphini) were provided by BioNostrum Pest Control Company^®^ (San Vicente del Raspeig, Spain). The larvae were fed with aphids (*Aphis hederae* Kaltenbach, 1843 and *Aphis fabae* Scopoli, 1763) until adult emergence. Third-stage larvae (L3) were used for chromosome preparations. Their brains were dissected in phosphate-buffered saline (PBS) and incubated in a colchicine solution (0.04% in NaCl 0.9%) for 30 min, before immersion in distilled water for 45 min to induce an osmotic shock, and were then preserved in an absolute ethanol/glacial acetic acid solution (3:1).

For the chromosome preparations, the brains were macerated in a 50% glacial acetic acid solution. The resulting suspension was then dispersed in droplets onto a glass slide and then dried on a heating plate at 42 °C. Afterward, the slides were dehydrated through a graded ethanol series (70%, 80%, and 100%, each for 30 s) and stored at −20 °C until further use. Chromosome spreads were either stained with Giemsa or mounted with VECTASHIELD containing the DAPI (4′-6-diamino-2-phenylindole) fluorochrome (Vector Labs, Burlingame, CA, USA) and examined under an Olympus BX51 fluorescence microscope (Olympus, Hamburg, Germany) equipped with an Olympus DP70 camera. Image acquisition and processing were performed using DP Manager (Olympus) and Adobe Photoshop CS4 software (Adobe Systems, San Jose, CA, USA). Chromosome morphology was determined on the basis of arm ratio, as proposed by Levan et al. [27].

### 2.2. C-Banding

Heterochromatin blocks were visualized using the C-banding technique, following a modified version of the protocol described by Sumner [28]. The slides were first treated with a 0.2 M hydrochloric acid solution for 10 min at 25 °C. They were then incubated in a 5% barium hydroxide solution at 60 °C for 1 min and 30 s, followed by washing with water. After a brief rinse in the initial hydrochloric acid solution, the slides underwent a final wash in 2 × SSC at 60 °C for 2 min. Finally, the slides were mounted using VECTASHIELD with DAPI.

### 2.3. DNA Extraction, Probes, and Fluorescence In Situ Hybridization (FISH)

The heads and thoraxes of a pool of five males were used for genomic DNA (gDNA) extraction, employing the NucleoSpin Tissue kit (Macherey-Nagel GmbH & Co., Düren, Germany).

The chromosomal localization of ribosomal DNA (rDNA) was determined by FISH. A fragment of the 18S rDNA was amplified using the primers 18S-965 and 18S-1573R [29], using *S. rueppellii* gDNA as a template. The resulting PCR product was labeled with biotin-16-dUTP (Roche Diagnostics GmbH, Mannheim, Germany) through nick translation, utilizing a DNA Polymerase I/DNase I mix (Invitrogen, San Diego, CA, USA). The labeled DNA was then precipitated and dissolved in a hybridization solution (50% *v*/*v* deionized formamide, 10% *v*/*v* dextran sulfate, 2 × SSC) to a final concentration of 15 ng/µL.

The fluorescence in situ hybridization (FISH) experiments were performed following the protocol described by Cabral-de-Mello and Marec [30]. Prior to hybridization, the slides were treated with RNase A (100 μg/mL in 2 × SSC) for 60 min at 37 °C and then washed in 2 × SSC. Subsequently, the slides were incubated in a fixation solution containing 3.7% formaldehyde in 4 × SSC, 0.1% *v*/*v* Tween-20, and 1% *w*/*v* skimmed milk, followed by dehydration in an ethanol series (70%, 90%, and 100%) for 5 min at each concentration. The hybridization solution was denatured at 95 °C for 10 min and then placed on ice for 3–5 min. Hybridization was carried out by applying 25 μL of probe solution to each slide. The slides were heated at 70 °C for 1 min and 30 s and subsequently transferred to a humid chamber for overnight incubation at 37 °C. After hybridization, the coverslips were removed and the slides were washed three times in 2 × SSC at room temperature. Before immunological detection, the slides were incubated for 15 min in Washing/Blocking Buffer (WBB; 4 × SSC, 0.1% *v*/*v* Tween-20, 1% *w*/*v* skimmed milk). The biotin-labeled probes were detected using Alexa Fluor 488-conjugated streptavidin (Invitrogen) at a concentration of 10 μg/mL in WBB. After incubation for 60 min at 37 °C, the slides were washed three times in WBB at room temperature, air-dried, and mounted with VECTASHIELD containing DAPI.

### 2.4. Mitogenomic Sequencing and Assembly Strategies

Approximately 4–5 μg of gDNA was sent to Novogen Company Ltd. (Cambridge, UK) for sequencing using the Illumina^®^ Hiseq™ 2000 platform (San Diego, CA, USA). A 350 bp fragment library was constructed, and 151 bp paired-end reads were generated, yielding around 2.6 gigabases (Gb) of sequencing data. To maintain high-quality data, low-quality sequences were removed using Trimmomatic v0.36 [31].

The de novo assembly of the mitogenome was performed with NOVOPlasty v4.3.1 [32], a tool designed for reconstructing organelle genomes from NGS data by extending a seed sequence. In this study, the *Sphaerophoria philanthus* Meigen, 1822 *cox1* gene (GenBank accession number OM372559) served as the seed for mitogenome assembly. Different K-mer values were tested, with 33 providing the most complete mitogenome assembly.

### 2.5. Mitogenome Annotation and Sequence Analysis

The mitogenome was annotated following the methodology described by Cameron [19], using the MITOS2 web server within the Galaxy platform (accessible at: https://usegalaxy.eu/?tool_id=toolshed.g2.bx.psu.edu%2Frepos%2Fiuc%2Fmitos2%2Fmitos2%2F2.1.9%2Bgalaxy0&version=latest, accessed on 31 March 2025). The annotation of the protein-coding genes (PCGs) was manually refined to ensure the correct identification of the start and stop codons and open reading frames, as well as consistency with other Syrphidae mitogenomes using Geneious R11.1.5 (Biomatters Ltd., Auckland, New Zealand). The annotation of ribosomal RNA (rRNA) genes remains one of the most challenging aspects of mitochondrial genome characterization [19]. In this case, the boundaries of the rRNA genes were extended until adjacent tRNA genes were identified, as per the standard methodology [33]. The large ribosomal subunit (*lrRNA*) was found between the *tRNA-Leu* and *tRNA-Val* genes, and the entire nucleotide stretch between them was designated as part of the *lrRNA* gene. Based on this method, the 3′ boundary of the small subunit rRNA (*srRNA*) was determined by the location of the *tRNA-Val* gene. However, there is no tRNA immediately upstream (5′ end) of the *srRNA* gene, leading to ambiguity in defining its starting point. To improve their accuracy, researchers have utilized secondary structure modeling and the identification of conserved sequence motifs at the 5′ end [19,34]. Because the MITOS annotation tool incorporates secondary structure into its predictions [19], its output was used as the basis for annotating the *srRNA* gene in species of *Sphaerophoria* Lepeletier & Serville, 1828.

Additionally, base composition analysis, mitogenome circularization, and secondary structure predictions were performed using the same software. Codon usage analysis was conducted in MEGA v.11.0.13. The assembled mitogenome with the corresponding annotations was submitted to GenBank under accession number PV660707.

### 2.6. Comparative Phylogenetics

The available Syrphinae mitogenomes were retrieved from GenBank, comprising a dataset of 80 sequences representing 60 species (Supplementary Table S1). Multiple sequences from the same species were included due to variations in their origins and sizes. In GenBank, mitogenome sequences were available for only two *Sphaerophoria* species: *S. philanthus* and *S. taeniata* Meigen, 1822. The mitogenome of *S. philanthus* (NC_071899) is annotated, whereas that of *S. taeniata* (OX016545) is not. To facilitate comparisons, the genome of *S. taeniata* was annotated, and the annotation of *S. philanthus* was reviewed using the same criteria applied to *S. rueppellii*.

Additionally, as an external outgroup, we included the mitochondrial genome sequences of five Syrphidae species belonging to the subfamily Eristalinae, which is phylogenetically close to the subfamily Syrphinae [5,35] (Supplementary Table S1).

The concatenated PCGs were aligned using MAFFT v7.453 software [36]. Poorly aligned positions and divergent regions were removed using the Gblocks program v.0.91.1 [37] (available at https://ngphylogeny.fr/tools/tool/276/form, accessed on 13 April 2025). Phylogenetic relationships were inferred through the Maximum Likelihood (ML) partitioned model [38] in IQtree2 [39]. We used the GTR + F + I + G4 model as it showed the lowest Bayesian information criterion (BIC) calculated with ModelFinder [40] with 1000 ultrafast bootstrap [41] replicates to assess branch support.

## 3. Results and Discussion

### 3.1. Cytogenetic Analysis

**Figure 1:**
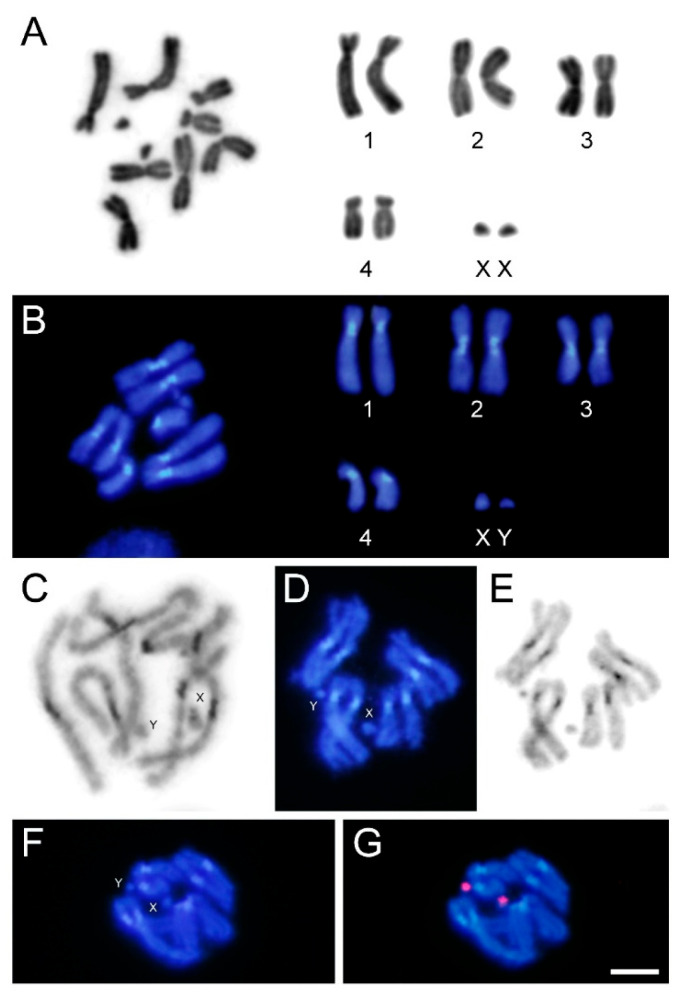
*Sphaerophoria rueppellii*. (**A**) Female mitotic metaphase and karyotype stained with Giemsa. (**B**) Male mitotic metaphase and karyotype stained with DAPI. (**C**) Male mitotic prometaphase stained with DAPI and inverted to grayscale. Male mitotic metaphase stained with DAPI (**D**), and same metaphase after C-banding and staining with Giemsa (**E**), showing coincidence of heterochromatic blocks with DAPI-positive pericentromeric region of autosomes. (**F**) DAPI staining and (**G**) FISH of male mitotic chromosomes using rDNA as probe, showing presence of hybridization signals (in red) on both sex chromosomes. Bar = 5 µm.

The karyotypic analysis shows that the analyzed *S. rueppellii* specimens have a chromosome number of 2n = 10. The karyotype consists of four pairs of large autosomes and one heteromorphic pair of small chromosomes corresponding to the sex chromosomes (Figure 1A,B). The largest autosomes (pair 1) and the smallest ones (pair 4) are submetacentric, while the intermediate-sized autosomes (pairs 2 and 3) are metacentric. Regarding the sex chromosomes, the X is larger than the Y, and their morphologies cannot be determined in mitotic metaphases due to their small size. However, in prometaphases, where the chromosomes are less condensed, it is possible to observe that the X chromosome is clearly acrocentric (Figure 1C). Even in this phase, the small size of the Y chromosome does not allow for a clear determination of its morphology.

Within the Syrphidae family, the chromosomal number and sex chromosome system have been determined for over 700 taxa, primarily due to the extensive work conducted by J. Wallace Boyes, Janny M. van Brink, and their collaborators [42,43,44,45,46,47,48,49,50,51]. In the past 45 years, only nine additional species have been analyzed [52,53,54]. The analyzed species exhibit diploid numbers ranging from 2n = 8 to 2n = 14 and possess an XY sex chromosome system. Within the genus *Sphaerophoria* Lepeletier & Serville, 1828, approximately twenty species have been studied, all of which have a chromosomal number of 2n = 8 [55]. However, chromosomal polymorphisms have been identified in some of them, leading to higher chromosomal numbers [49].

**Figure 2:**
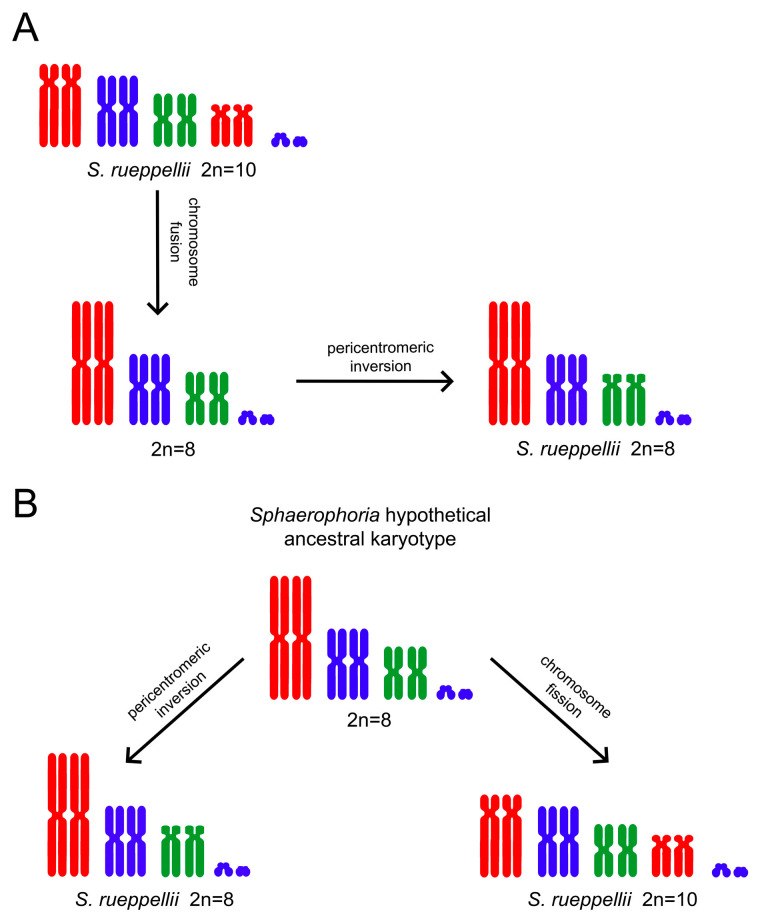
*Sphaerophoria rueppellii* karyotypes. (**A**) Karyotype evolution in *S. rueppellii* according to Boyes et al. [49]. (**B**) Alternative hypothesis. Possible explanation provided in text.

In S. *rueppellii*, populations exhibiting chromosomal counts of 2n = 8 and 2n = 10 have been documented [49]. Populations with 2n = 8 have been identified in northern Italy and the Netherlands, whereas those with 2n = 10 have been reported in northern Italy, Sicily, Portugal, and Ethiopia. In the latter karyotype, the medium-sized chromosomes are metacentric, while the largest and smallest pairs are submetacentric to subtelocentric (Figure 2). Additionally, a population from Biella (Italy) presented individuals with 2n = 12 chromosomes. This karyotype is similar to that of the 2n = 8 individuals, but includes two pairs of microchromosomes of unknown origin [49].

The material analyzed in this study exhibits a karyotype similar to the 2n = 10 configuration described by Boyes et al. [49]. Those authors proposed that the 2n = 8 karyotype arose from 2n = 10 by means of an end-to-end fusion of submetacentric–subtelocentric autosomes, resulting in the formation of a metacentric chromosome (Figure 2A). Subsequently, a pericentric inversion in one of the autosomes would have led to the metacentric chromosome observed in the 2n = 8 karyotype. An alternative explanation could account for the chromosomal polymorphism observed in S. *rueppellii*. The intermediate karyotype proposed by Boyes et al. [49], in which all autosomes are metacentric, was not found in any of the analyzed populations of this species. However, this karyotype is the most common among the *Sphaerophoria* species studied to date. It is therefore plausible to consider this configuration as the ancestral karyotype within the genus, including *S. rueppellii*. The karyotypes observed in *S. rueppellii* could have easily derived from this ancestral form (Figure 2B). The 2n = 8 karyotype may have arisen through a pericentric inversion in one autosome, converting it into a submetacentric–subtelocentric chromosome. The centric fission of the largest metacentric chromosome in the ancestral karyotype could have produced the 2n = 10 configuration.

**Figure 3:**
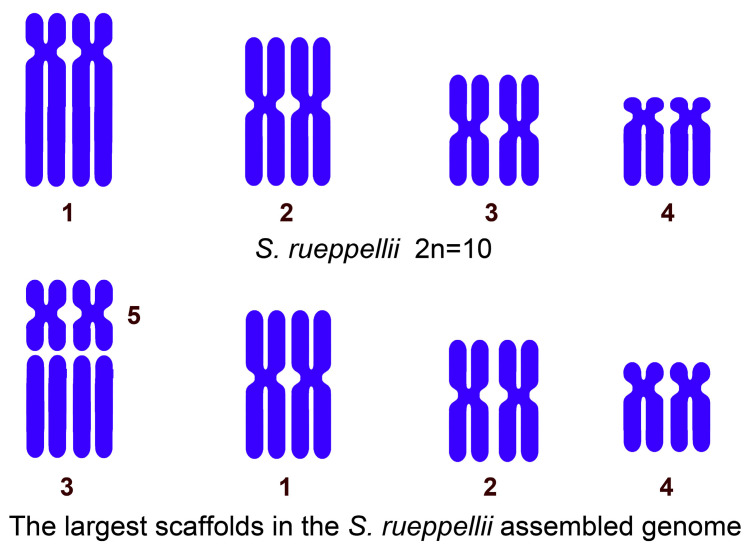
A comparison between the autosomes in the 2n = 10 karyotype and the five largest near-chromosome-level scaffolds (autosomes) obtained from the *S. rueppellii* genome assembly (GCA_920937365.1).

A recent high-quality, near-chromosome-level de novo genome assembly was generated for *S. rueppellii*, using a hybrid approach that combined PacBio long-read and Illumina short-read sequencing data, followed by super-scaffolding with Hi-C data [56]. This process yielded six chromosome-level scaffolds, suggesting the presence of five pairs of autosomes plus a small sex chromosome (likely the X chromosome), corresponding to a chromosomal number of 2n = 12. However, a karyotype with five pairs of autosomes has not been observed in any of the *S. rueppellii* populations analyzed to date. It is possible that the *S. rueppellii* individuals used for genome sequencing actually possess only four pairs of autosomes (2n = 10). This hypothesis is based on several observations: the relative sizes of the six largest scaffolds and the chromosome sizes reported by Boyes et al. [49]. Furthermore, scaffold 3 (pseudochromosome 3) does not show a clearly defined centromere. Bailey et al. [56] consider that this is likely due to an incorrect assembly. In addition, scaffold 5 appears to correspond to a small metacentric chromosome not previously identified in *S. rueppellii* (Figure 3). We suggest that scaffold 5 contains the centromere of the largest autosome, while scaffold 3 corresponds to its long arm. This is why a centromere could not be detected in scaffold 3 in the genome assembly. Furthermore, there is a correlation between the relative sizes of the autosomes and the sizes of the scaffolds (Table 1). For instance, chromosome 4 accounts for between 15.9% and 18.0% of the total genome length. This percentage is very close to that of scaffold 4 (16.5%) in relation to the assembled genome (455 Mb). This putative inconsistency between pseudochromosomes obtained from genome assembly and the chromosome number observed reinforces the importance of the complementary analysis of karyotype in genome assembly projects.

Staining with DAPI reveals the presence of prominent DAPI-positive chromatin blocks in the pericentromeric regions of all autosomes (Figure 1B–D). C-banding confirms the presence of heterochromatin blocks located in the same pericentromeric regions, coinciding with the DAPI + signals (Figure 1E). Both sex chromosomes appear to be euchromatic.

Although the chromosome number and sex chromosome system are known for over 700 taxa within the Syrphidae family, C-banding techniques have been applied to only a limited number of species, albeit from two different subfamilies: Eristalinae and Syrphinae [52,53,54]. Despite the scarce data available, the presence of pericentromeric heterochromatin blocks in autosomes appears to be a common feature. However, the presence or absence of heterochromatin in the sex chromosomes is more variable, ranging from heterochromatin in both sex chromosomes (in some cases, completely heterochromatic), to heterochromatin in only one sex chromosome, or its absence from both. Among the species previously studied is *Sphaerophoria scripta* Linnaeus, 1758, which displays pericentromeric heterochromatin in all autosomes and the X chromosome, while its Y chromosome is euchromatic [53]. In *S. rueppellii*, the DAPI+ heterochromatin observed in its autosomes corresponds to pericentromeric heterochromatin. In the X chromosome, a DAPI + pericentromeric region is also visible (Figure 1C), but this region does not produce differential staining after C-banding (Figure 1E).

FISH with the rDNA probe revealed clusters located on both sex chromosomes (Figure 1F,G). To our knowledge, no previous studies have addressed the localization of rDNA genes in Syrphidae. However, their position has been determined in other dipteran groups, with varying results. While it is generally common for major rDNA to be located on the sex chromosomes, there are also groups with additional clusters on autosomes, and even some cases where rDNA is exclusively located on autosomes [57,58,59]. Further analysis of related species will be necessary to determine whether a specific pattern for rDNA localization is conserved in Syrphidae.

### 3.2. Mitogenome Analysis, Gene Organization, and Sequence Analysis

**Figure 4:**
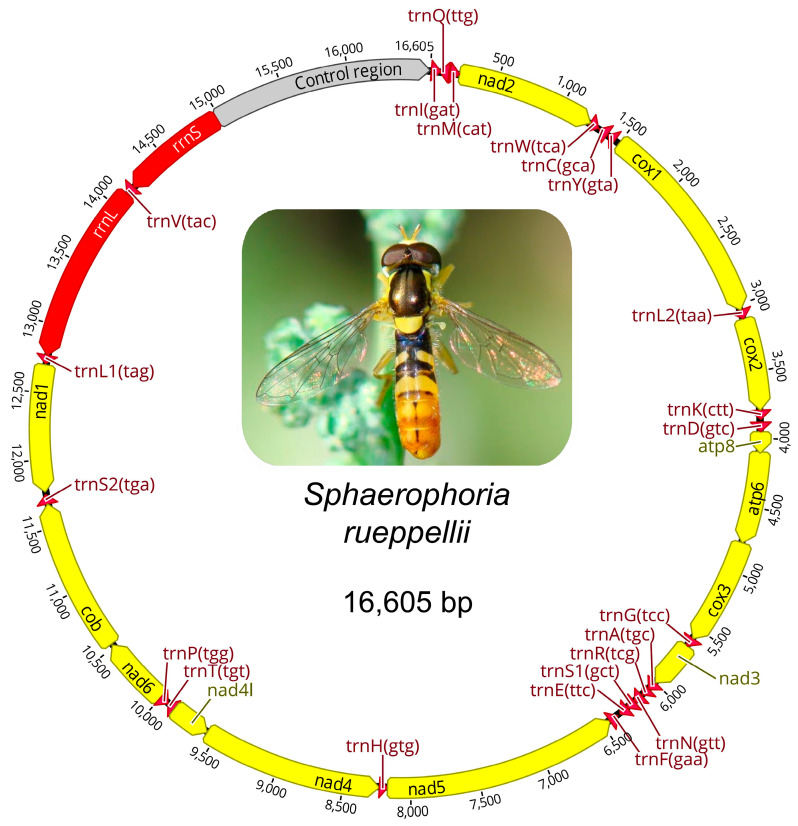
Graphical map of mitogenome of *Sphaerophoria rueppellii*.

The mitogenome of *S. rueppellii* is 16,605 bp in length (Figure 4). GenBank currently contains mitogenome sequences for only two other species of the *Sphaerophoria* genus, with sizes similar to that found in *S. rueppellii*: *S. taeniata* (16,422 bp) [60] and *S. philanthus* (16,036 bp). These sizes fall within the range of other species analyzed within the tribe Syrphini, with mitogenomes ranging from 15,326 bp in *Eupeodes corollae* Fabricius, 1794 [61] to 19,366 bp in *Chrysotoxum bicinctum* Linnaeus, 1758 [62] (Supplementary Table S1). The *S. rueppellii* mitogenome comprises the standard complement of mitochondrial genes: 13 protein-coding genes (PCGs), 2 rRNA genes, 22 tRNA genes, and a non-coding control region rich in adenine and thymine (A + T) (Table 2, Figure 4). The annotation of the mitogenomes of *S. rueppellii*, as well as *S. philanthus* and *S. taeniata*, is provided in Supplementary Table S2. The analysis of mitogenomes across various arthropod groups has allowed for the establishment of the gene order in the ancestral pancrustacean–insect genome [63,64]. There are no gene rearrangements in the mitogenome of *S. rueppellii* compared to this ancestral mitogenome, nor in the other two *Sphaerophoria* species.

In *S. rueppellii*, four protein-coding genes (*nad5*, *nad4*, *nad4l*, and *nad1*) are located on the light (L) strand, while the remaining nine are located on the heavy (H) strand (Table 2). Translation initiation in these genes predominantly occurs with typical ATN start codons, except for *cox1* and *nad1*, which start with the TTG codon. These same start codons are found in all three *Sphaerophoria* species (Supplementary Table S2). In *S. rueppellii*, as well as in *S. taeniata*, ten of the thirteen protein-coding genes end with a complete TAA stop codon, whereas incomplete stop codons (T– or TA–) are found in *nad2*, *cox1*, and *nad5*. In *S. philanthus*, in addition to these three genes, the cob gene also ends with an incomplete stop codon (Supplementary Table S2). Truncated stop codons often result from gene sequences terminating just before the start of a downstream tRNA gene, with the complete stop codon restored post-transcriptionally through polyadenylation [33].

The mitogenome of *S. rueppellii* displays a strong A + T bias, with an A + T content of 80.77%, such as in other Syrphidae species [65], that is typical of insect mitogenomes [19,20,66]. The codon usage also reflects this bias towards A + T codons, with a clear preference for codons rich in A and T. To assess codon usage bias, Relative Synonymous Codon Usage (RSCU) was employed (Table 3). RSCU quantifies how frequently a specific codon is used compared to its expected frequency if all synonymous codons for an amino acid were utilized equally. An RSCU value of 1 indicates no bias, values greater than 1 suggest a codon is used more frequently than expected, and values less than 1 imply under-representation. RSCU is strongly influenced by the nucleotide composition of the genome. In mitochondrial genomes with high A + T contents, such as that of *S. rueppellii*, codons rich in adenine and thymine (e.g., NNA or NNT) exhibit elevated RSCU values, indicating their preferential usage. The most used codons are A + T-rich: TTA (Leu, 14.33%), ATT (Ile, 10.00%), TTT (Phe, 8.66%), and ATA (Met, 7.62%). As a result, the amino acids these codons encode are particularly abundant in the mitochondrial proteome of this species. The A + T bias also extends to termination codons. Within the protein-coding genes (PCGs), TAA, along with its incomplete forms (TA–, T–), is the only stop codon used, appearing 13 times, while TAG is entirely absent.

**Figure 5:**
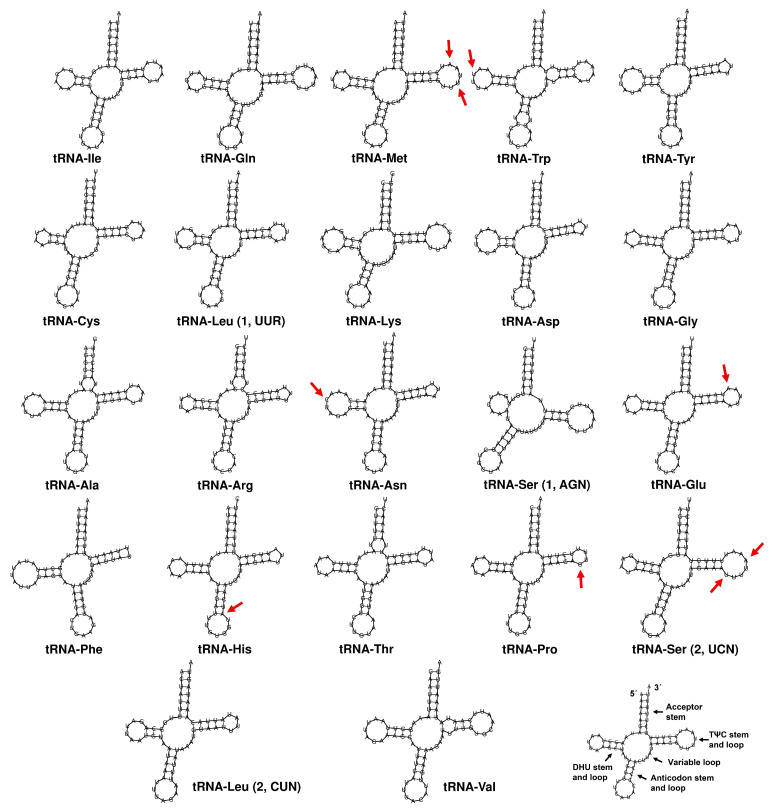
Secondary structures of all tRNAs in *Sphaerophoria rueppellii*. Arrows indicate variable positions in comparison among three *Sphaerophoria* species.

Figure 5 shows the 22 tRNA genes identified in *S. rueppellii*, with lengths ranging from 64 bp (*tRNA-Arg*) to 71 bp (*tRNA-Lys*, *tRNA-Val*). Most of these tRNAs exhibit the expected cloverleaf secondary structure. However, an exception is found in *tRNA-Ser1* (AGN), which lacks a stable DHU arm, a trait commonly reported in many insects and other metazoans [67]. The tRNA sequences are highly conserved among the three *Sphaerophoria* species. In fact, no changes were found in 15 out of the 22 tRNAs. The observed mutations, which affect one or two positions, are located in the loops of the DHU and TΨC arms, regions where such variations are less likely to impact tRNA structure [68]. The only mutation observed outside these regions occurs in the stem of the anticodon arm of *tRNA-His*; however, this is unlikely to disrupt stability as it results in a U–A to U–G base pair change (or vice versa), which probably does not significantly affect arm pairing stability.

Mitochondrial genomes typically contain two classes of non-coding elements: the control region (CR) and intergenic spacers (IGSs). *S. rueppellii* features 17 IGSs in addition to its CR (Table 2). The most extensive IGS, 41 bp long, is located between the *tRNA-Tyr* and *cox1* genes, while the remaining spacers range from 1 to 19 bp in length. Four gene overlaps are present in the *S. rueppellii* mitogenome: between the *tRNA-Ile* and *tRNA-Gl*n genes (3 bp), *atp8* and *atp6* (7 bp), *tRNA-Ala* and *tRNA-Arg* (1 bp), and between nad4 and *nad4l* (7 bp). The same overlaps, and with identical lengths, are found in the mitogenomes of other *Sphaerophoria* species (Supplementary Table S2). The *atp8*–*atp6* overlap, which includes the shared sequence ATGATAA, contains the ATG start codon of *atp6* and the TAA stop codon of *atp8*. The *nad4l*–*nad4* overlap, with the sequence ATGTTAA (in the L strand), includes the ATG start codon of *nad4* and the TAA stop codon of *nad4l*. These overlaps are known to be conserved features in arthropods [19,69], although some of them are absent in certain hymenopteran groups [70].

In mitochondrial genomes, the CR typically represents the largest segment of non-coding DNA. Located immediately downstream of the small subunit rRNA (*srRNA*) gene, this region is believed to play a key role in the regulation of both replication and transcription processes [71]. Its base composition and length vary widely among species [66,72]. In Syrphidae mitogenomes, the CR exhibits considerable variability and is one of the main contributors to the overall mitogenome length [65,66]. Among *Sphaerophoria* species, differences in CR length are also evident, with *S. rueppellii* showing the longest control region (1633 bp), compared to *S. philanthus* (1059 bp) and *S. taeniata* (1435 bp) (Supplementary Table S2).

Despite differences in overall length, the CR sequences of the three *Sphaerophoria* species exhibit a high degree of conservation at both the 5′ and 3′ ends (Supplementary Figure S1). The increased size of the CR in *S. rueppellii* is due to the presence of a tandemly repeated element with four identical copies of a 215 bp sequence. Tandem repeat structures within the mitochondrial CR have been observed across several insect orders, and as observed in *S. rueppellii*, with a high degree of sequence conservation [66,73,74,75]. Similar sequences were also identified in the CRs of *S. taeniata* (221 bp) and *S. philanthus* (222 bp), with >84% sequence identity to the repeat unit found in *S. rueppellii*. However, in these two species, the repeated element is not organized in tandem. In *S. philanthus*, the 221 bp sequence is present as a single copy, while in *S. taeniata*, two copies are present, separated by a 154 bp spacer region that partially resembles the repeat. The two 222 bp sequences in *S. taeniata* share 98.64% sequence identity with one another. Notably, these sequences exhibit greater similarity to the *S. philanthus* copy (93.67% and 94.12%) than to that of *S. rueppellii*, potentially suggesting a closer phylogenetic relationship between *S. taeniata* and *S. philanthus*.

In the published genome assembly of *S. rueppellii* [56], the mitochondrial genome was also assembled and reported to be 16,387 bp in length, which differs from the 16,605 bp observed in our assembly. Unfortunately, that publication does not provide any accession number for the mitochondrial sequence, nor does it appear to be deposited in GenBank, making it impossible to determine the exact cause of the size discrepancy. However, the difference in length between the two assemblies, 218 bp, is very close to the 215 bp length of the tandem repeat identified in the CR of our assembly, suggesting that the mitogenome reported by Bailey et al. [56] may contain only three copies of the repeat unit, as opposed to the four observed in our data. Although this difference might also be attributable to the use of distinct assembly methodologies, another plausible explanation is the existence of heteroplasmy in mitogenome size, caused by intraspecific variation in the number of repeat units within the CR. Such heteroplasmy variation has been documented in other insect taxa [66,76,77].

### 3.3. Phylogenetic Analyses

**Figure 6:**
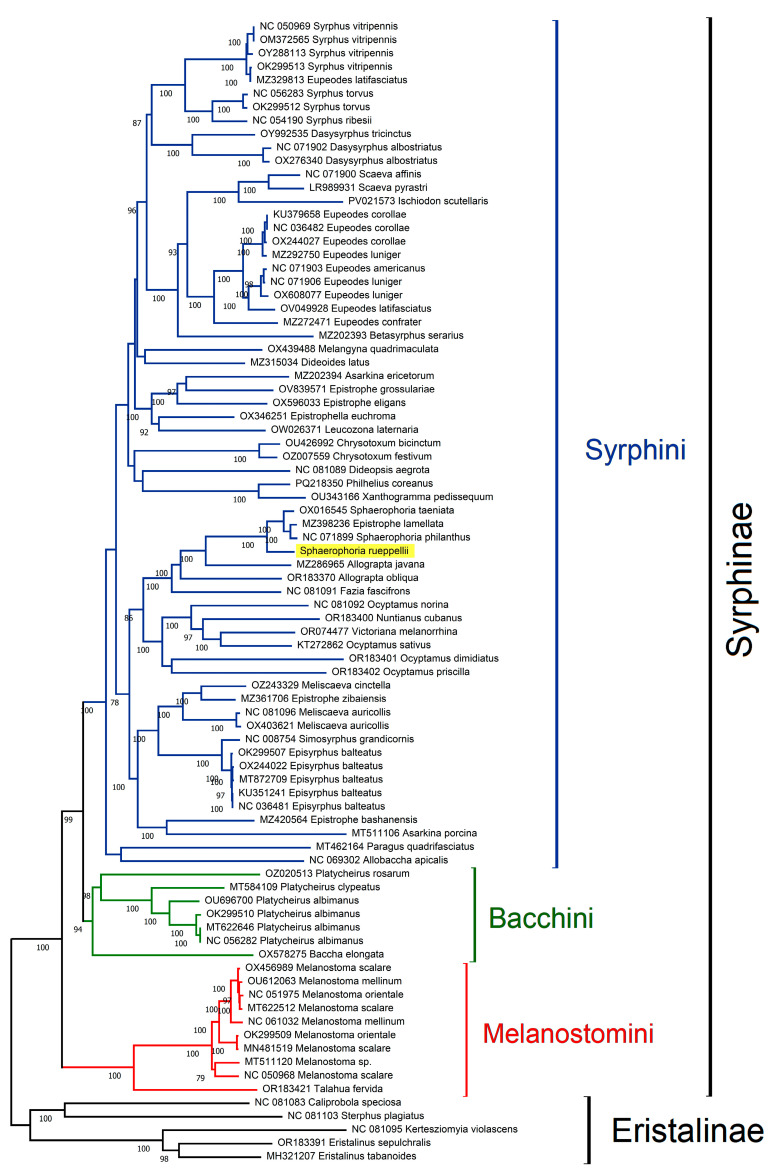
Phylogenetic relationships based on Maximum Likelihood (ML) analysis. Bootstrap values above 70 shown next to branches. *S. rueppellii* highlighted in yellow.

The classification of the subfamily Syrphinae into tribes has undergone numerous revisions. Recent studies using coding loci [35] support the division of the subfamily into three distinct tribes: Syrphini, Bacchini, and Melanostomini. For phylogenetic analysis, available data from the GenBank database were used for all the currently represented Syrphinae species. The Maximum Likelihood (ML) tree, based on concatenated PCG sequences, is shown in Figure 6. This analysis clusters Syrphinae sequences into three well-supported clades, with a topology similar to that obtained by Mengual et al. [35], thus supporting the division of the subfamily Syrphinae into three tribes.

The genus *Sphaerophoria* belongs to the tribe Syrphini. The phylogenetic reconstruction of Syrphini species based on mitochondrial genome data reveals that the majority of genera appear to form coherent, well-supported clades, which generally correspond with current taxonomic classifications and support their monophyletic status. However, exceptions exist, and the results also highlight some unresolved taxonomic issues, potential misidentifications, and cases of paraphyly or polyphyly.

A primary observation is that most species from the same genus tend to group together, suggesting that they share a common ancestor and supporting the monophyly of those genera. Genera such as *Eupeodes* Osten Sacken, 1877, *Scaeva* Fabricius, 1805, *Syrphus* Fabricius, 1775, *Dasysyrphus* Enderlein, 1938, and *Chrysotoxum* Meigen, 1803 form distinct and well-resolved clades, consistent with expectations from morphology-based taxonomy.

One illustrative case that underscores the importance of data quality and accurate specimen identification is that of *Eupeodes latifasciatus* Macquart, 1829. Two mitochondrial genome sequences labeled under this species are available in GenBank. One of them (acc. no. OV049928) clusters as expected within the *Eupeodes* clade, alongside *E. luniger* Meigen, 1822 and *E. americanus* Wiedemann, 1830. However, the second *E. latifasciatus* sequence (acc. no. MZ329813) is placed within the *Syrphus* clade, closely related to *S. vitripennis* Meigen, 1822. This suggests a likely misidentification, with the misassigned sequence perhaps belonging to a *Syrphus* species. This example highlights the critical need for the careful taxonomic validation and curation of sequences submitted to public repositories such as GenBank.

Beyond individual misidentifications, the tree reveals deeper evolutionary patterns. Some genus-level groupings appear to be paraphyletic, such as the genera *Ocyptamus* Macquart, 1834 and *Victoriana* Miranda, 2020, both with Neotropical distribution, as well as *Allograpta* Osten Sacken, 1875 and *Sphaerophoria*. Adults and immature stages of the species belonging to the *Allograpta* and *Sphaerophoria* genera also exhibit a significant similarity in their morphological characteristics [11,78]. These findings are consistent with previous phylogenetic studies [79,80,81], which also identified complex relationships among these genera.

A particularly interesting and problematic case involves the genus *Epistrophe* Walker, 1852, which appears polyphyletic in the tree. Species attributed to *Epistrophe* are distributed across multiple clades, rather than forming a cohesive monophyletic group. This pattern had already been noted by Li et al. [65], and is further confirmed in the present analysis. The taxonomic instability of Epistrophe points to the need for comprehensive revisions incorporating molecular, morphological, and ecological data.

Within the *Sphaerophoria* clade, an unexpected placement occurs with *Epistrophe lamellata* Huo, Ren et Zheng, 2007. This species is nested within the *Sphaerophoria* group rather than with other *Epistrophe* species. The presence of *E. lamellata* in this clade raises two possibilities: it could be a case of mislabeling or incorrect identification, or it may reflect a deeper issue with the species’ generic assignment. Unfortunately, due to limited information available for *E. lamellata*, it is difficult to determine the cause with confidence. However, this pattern is not entirely novel because *E. lamellata* was also placed near *Sphaerophoria* in a previous tree published by Guo et al. [82], though no explanation for this was provided. In another phylogenetic reconstruction by Li et al. [65], *E. lamellata* appeared to be closely related to *Allograpta javana* Wiedemann, 1824, suggesting its placement near *Allograpta* + *Sphaerophoria*.

In summary, the phylogenetic analysis confirms the monophyly of several Syrphini genera while also revealing unexpected placements and possible paraphyly in others. These findings emphasize the importance of accurate species identification, proper annotation in genetic databases, and the continued integration of genomic data with traditional taxonomy. Particularly for complex groups like *Epistrophe*, a revision that incorporates broader sampling and multi-gene approaches may be necessary in order to resolve their evolutionary relationships definitively.

## Figures and Tables

**Table 1:** A comparison between the relative length of each autosome in the 2n = 10 karyotype of *Sphaerophoria rueppellii* [49] and the relative size of the largest scaffolds obtained from its genome assembly [56].

	Chromosome 1	Chromosome 2	Chromosome 3	Chromosome 4
The relative length of each autosome in the 2n = 10 karyotype.	27.9–28.8%	25.5–27.8%	22.0–24.5%	15.9–18.0%
	Scaffolds 3 + 5	Scaffold 1	Scaffold 2	Scaffold 4
The percentage of each scaffold relative to the total assembled megabases.	31.9% (19.14 + 12.73)	27.6%	22.3%	16.5%

**Table 2:** Annotation of complete mitogenome of *Sphaerophoria rueppellii*. IGN: Intergenic nucleotides. Negative values refer to overlapping nucleotides.

Gene	Strand	Nucleotide Number		Length	IGN	Start Codon	Stop Codon
*(I) tRNA-Ile*	H	1	66	66	−3		
*(Q) tRNA-Gln*	L	64	132	69	3		
*(M) tRNA-Met*	H	136	204	69	0		
*nad2*	H	205	1237	1033	0	ATT	T–
(W) *tRNA-Trp*	H	1238	1306	69	16		
(C) *tRNA-Cys*	L	1323	1388	66	6		
(Y) *tRNA-Tyr*	L	1395	1460	66	41		
*cox1*	H	1502	3035	1534	0	TTG	T–
(L1) *tRNA-Leu* (UAA)	H	3036	3101	66	4		
*cox2*	H	3106	3789	684	0	ATG	TAA
(K) *tRNA-Lys*	H	3790	3860	71	14		
(D) *tRNA-Asp*	H	3875	3944	70	0		
*atp8*	H	3945	4106	162	−7	ATT	TAA
*atp6*	H	4100	4777	678	7	ATG	TAA
*cox3*	H	4785	5573	789	3	ATG	TAA
(G) *tRNA-Gly*	H	5577	5642	66	0		
*nad3*	H	5643	5996	354	2	ATT	TAA
(A) *tRNA-Ala*	H	5999	6067	69	−1		
(R) *tRNA-Arg*	H	6067	6130	64	14		
(N) *tRNA-Asn*	H	6145	6211	67	0		
(S1) *tRNA-Ser* (UCU)	H	6212	6278	67	1		
(E) *tRNA-Glu*	H	6280	6346	67	19		
(F) *tRNA-Phe*	L	6366	6433	68	0		
*nad5*	L	6434	8169	1736	0	ATG	TA–
(H) *tRNA-His*	L	8170	8234	65	0		
*nad4*	L	8235	9575	1341	−7	ATG	TAA
*nad4l*	L	9569	9865	297	2	ATG	TAA
(T) *tRNA-Thr*	H	9868	9933	66	0		
(P) *tRNA-Pro*	L	9934	9999	66	2		
*nad6*	H	10,002	10,526	525	11	ATT	TAA
*cob*	H	10,538	11,674	1137	3	ATG	TAA
(S2) *tRNA-Ser* (UGA)	H	11,678	11,746	69	16		
*nad1*	L	11,763	12,710	948	1	TTG	TAA
(L2) *tRNA-Leu* (UAG)	L	12,712	12,776	65	0		
*lrRNA*	L	12,777	14,111	1335	0		
(V) *tRNA-Val*	L	14,112	14,182	71	0		
*srRNA*	L	14,183	14,972	790	0		
Control Region		14,973	16,605	1633			

**Table 3:** Codon usage of *Sphaerophoria rueppellii* mitogenome protein-coding genes. Total of 3741 codons analyzed. RSCU: Relative Synonymous Codon Usage. * = termination codon.

Codon	n	RSCU	%	Codon	n	%	RSCU	Codon	n	%	RSCU	Codon	n	%	RSCU
UUU(F)	324	1.91	8.66	UCU(S)	124	3.31	3.06	UAU(Y)	184	4.92	1.96	UGU(C)	38	1.02	2
UUC(F)	16	0.09	0.43	UCC(S)	5	0.13	0.12	UAC(Y)	4	0.11	0.04	UGC(C)	0	-	0
UUA(L)	536	5.54	14.33	UCA(S)	96	2.57	2.37	UAA(*)	13	0.35	2	UGA(W)	96	2.57	1.96
UUG(L)	10	0.1	0.27	UCG(S)	0	-	0	UAG(*)	0	-	0	UGG(W)	2	0.05	0.04
CUU(L)	18	0.19	0.48	CCU(P)	81	2.17	2.53	CAU(H)	69	1.84	1.92	CGU(R)	20	0.53	1.43
CUC(L)	0	0	-	CCC(P)	6	0.16	0.19	CAC(H)	3	0.08	0.08	CGC(R)	0	-	0
CUA(L)	16	0.17	0.43	CCA(P)	40	1.07	1.25	CAA(Q)	70	1.87	1.97	CGA(R)	35	0.94	2.5
CUG(L)	0	0	-	CCG(P)	1	0.03	0.03	CAG(Q)	1	0.03	0.03	CGG(R)	1	0.03	0.07
AUU(I)	374	1.98	10.00	ACU(T)	85	2.27	1.85	AAU(N)	210	5.61	1.92	AGU(S)	42	1.12	1.04
AUC(I)	3	0.02	0.08	ACC(T)	2	0.05	0.04	AAC(N)	9	0.24	0.08	AGC(S)	2	0.05	0.05
AUA(M)	285	1.9	7.62	ACA(T)	97	2.59	2.11	AAA(K)	83	2.22	1.8	AGA(S)	55	1.47	1.36
AUG(M)	15	0.1	0.40	ACG(T)	0	-	0	AAG(K)	9	0.24	0.2	AGG(S)	0	-	0
GUU(V)	71	1.75	1.90	GCU(A)	88	2.35	2.32	GAU(D)	63	1.68	1.88	GGU(G)	41	1.10	0.79
GUC(V)	0	0	-	GCC(A)	3	0.08	0.08	GAC(D)	4	0.11	0.12	GGC(G)	0	-	0
GUA(V)	89	2.2	2.38	GCA(A)	60	1.60	1.58	GAA(E)	70	1.87	1.92	GGA(G)	159	4.25	3.07
GUG(V)	2	0.05	0.05	GCG(A)	1	0.03	0.03	GAG(E)	3	0.08	0.08	GGG(G)	7	0.19	0.14

## Data Availability

The *Sphaerophoria rueppellii* mitogenome sequence was submitted to NCBI (acc. number PV660707).

## Supplementary Materials

The following supporting information can be downloaded at: https://www.mdpi.com/article/10.3390/insects16060604/s1, Figure S1: Control region analysis; Table S1: Available mitogenomes from Syrphinae subfamily; Table S2: Mitogenome annotations in *Sphaerophoria* species.
