# Supplementary material for: Cytogenetic and Molecular Characterization of Sphaerophoria rueppellii (Diptera, Syrphidae)
Source: Insects. 2025 Jun 8;16(6):604. doi: 10.3390/insects16060604 (PMC12193307; doi:10.3390/insects16060604)
Supplement: Supplementary file 1 [file insects-16-00604-s001.zip › Supplementary Figure S1-Control Region.pdf]

Supplementary Figure S1. Control region analysis. (A) Sequences of the CR in the three *Sphaerophoria* species. The first nucleotides of the tandemly repeated sequence are highlighted in yellow, and the final nucleotides of the repeat are shown in green. (B) Alignment of the CRs. Each of the four tandem repeat copies in the CR of *S. rueppellii* is marked with a different color. (C) Alignment of the repeats of the CRs in the three *Sphaerophoria* species and percent identity matrix.

A

>S_rueppellii_CR
AAATAATTTAATCATTTATTATATAAAAATAATAAATAATTAATATATTTTTAAATAATATTAATTAGTAAATATAA

TTTTATAACTTATATTTTATTAAATAATTAAAATTAAGTAATATCTACCAGAAAAAAACTATTTTTTAATTAATATTTTATTTTATATTTAATATAATATTTATCCCCTAAATAAATAATATTTCTAATATAAAATTTTAAAAGATATTTAATAATAATAGAAAAAACTCAGTTTTATTTTTAAATTATAAAATTTAAAATTTTTGTTAATTTAG
TTTTATAACTTATATTTTATTAAATAATTAAAATTAAGTAATATCTACCAGAAAAAAACTATTTTTTAATTAATATTTTATTTTATATTTAATATAATATTTATCCCCTAAATAAATAATATTTCTAATATAAAATTTTAAAAGATATTTAATAATAATAGAAAAAACTCAGTTTTATTTTTAAATTATAAAATTTAAAATTTTTGTTAATTTAG
TTTTATAACTTATATTTTATTAAATAATTAAAATTAAGTAATATCTACCAGAAAAAAACTATTTTTTAATTAATATTTTATTTTATATTTAATATAATATTTATCCCCTAAATAAATAATATTTCTAATATAAAATTTTAAAAGATATTTAATAATAATAGAAAAAACTCAGTTTTATTTTTAAATTATAAAATTTAAAATTTTTGTTAATTTAG
TTTTATAACTTATATTTTATTAAATAATTAAAATTAAGTAATATCTACCAGAAAAAAACTATTTTTTAATTAATATTTTATTTTATATTTAATATAATATTTATCCCCTAAATAAATAATATTTCTAATATAAAATTTTAAAAGATATTTAATAATAATAGAAAAAACTCAGTTTTATTTTTAAATTATAAAATTTAAAATTTTTGTTAATTTAG

TTTTATAACTTATATTTTATTAAATAATTAAAATT

TATAAAATTATATAAATAAATTTATTAGATAATTTAAATAAGTAAATAATTTTATAAATTTTAATGATGATTTTTTTTTTTTTTTTTTTTTTTTTTTATATATATATATATATATATATAGATAATCTATATGTCTAAATATTAATATTTTTATAAATACAT
TAATATTATTTCAAAAAAAACCCTAATTTATATAAATGATGATTTAAATATAAATTAAAATCTATATTATTAAAAATAATATAATTAATGAAGGGTTTTACTTAATTATAGAAAGCAAAATTTAATTAAATATTATATATTTATGTTTAGTAATCATAT
ATTTAATAAATATATATATATATAATGTATATTTATTATATAATATAAATAATCGAGTTATATATATTATTTAAAATTTTATTTGATAAATATTTAATCTAAATTTTAAATTACATTTAAAAATATATTCTTGAATATAGTTTTTAAATGTAATTTAAAAT
TTATTAAATATTTATAGTTAAACATATAAATGTTTAATGGATACATTTTTATTTATATACAATACATTCGTACTAAATTAAATTATTTAATTTATATACCAATAATAATTTGATTTTACAAAAAAAAAAATTATTTTCCTAAAAATAATGAATTATTTA
ATATAATATATTTTTTTCATAAAAT

>S_taeniata_CR
AAATAATTTAATAATTTATAATATAAAAATAATAAATTATTAATATATTTTTAAATAATATTAATTAGTAATAAAAA

TTTTATAATTTATATTTTATTAAATAATTGATATTAAGTAATAGCTCCCGAAAAAAATCAACTATTTTTTTATTAAAATTCTATTTTATATTTAAATATATTATTTATCCCCTAAATAAATAATATTTCTAATTTAAAATTTAAAGAATATATAATAATAATTGAAAAAAAAACTCAGTTTTATTTTTAAATTTAAAAATTTTAAATTTTAATAAATTTAA
TATATAACTCATATTTTATTAAATAATTAAAATTAAGTACTAATTCCAAAAAAATAATCTATTTTTTCATTAAAATTTTATTTTATATTTAAATACATTATATATATATATATATATATATATATACTAAATAAATATTATTTATAAATTAAAATTTA
ATTTATAATTTATATTTTATTAAATAATTGATATTAAGTAATAGCTCCCGAAAAAAATCAACTATTTTTTTATTAAAATTCTATTTTATATTTAAATATATTATTTATCCCCTAAATAAATAATATTTCTAATTTAAAATTTAAAGAATATATAATAATAATTGAAAAAAAAACTCAGTTTTATTTTTAAATTTAAAAATTTTAAATTTTAATAAATTTAA

TATATAACTCATATTTTATTAAATAATTAAAATTAA

TATAAATTTTTATAAAATTTTTATACAATTAAATAATTTTAATAAGTAAATTTAAATATAAATTATTAAATTATATTAATAAATAAATTAGATAACTTAAATAAGTAAATGATTTTATAAAATTTATAAATTAAATTTTTTTTTTTTTTTTTTTTT
AATATATATATATATATATAGATAATCTATATGTCTAAATATTAATATTTTTATAAATACATTAATATTATTTCAAAAAAAACCCTAATTTATATAAATAATGATTTAAATATAAATTAAAATCTATATTATTAAAGATAATCTAATTATAGAAAGTTT
TTACTTAATTATGGAAAGCAATATTTAATTAAATATTATATCCTTATGTTTAATAATTGTATATTTAATAAATATATATATATATATATAATAAATATTTATTTTAATATAAATAATCGAGTTATATATATTTATTAAAATATTATTTGATAAATATTTAA
TGTAAATTTTAAATTACATTTAAAAATATATTCTTGAATATAGTTTTTAAATGTAATTTAAAATTTATTAAATATTTATAGTTAAACATATAAATGTTTAATGGATAAGTTTTTATTTATACACAACGCACTCATACTAAATTAAATTATTTAATTTAT
ATGTCAGTAATAATTTAGTTTTATAAAAAAATATTATTTTTTTCCTAAAAATAATAAATTATATGATAAAAGATATTTTTTTCATAAAAT

>S_philanthus_CR
AAATAATTTAATAATTTATAATATAAAAATAATAAATTATTAATATATTTTTAAATAATATTAATTAGTAATAAAAA

TTTTATAAATCATATTATATTAAATAATTATAATTAAGTAATARCTCCCGAAAAAAATCAACTATTTTTTTTATTAAAATTATATTTTATATTTAAATATATTATTTATCCCCTAAATAAATAATATTTCTAAATTAAAATTTAAATAATTTATAATAATAATTGAAAAAAAAACTCAGTTTTATTTTTAAATTTAWAAATTTTAAATTTTAATAAATTTAA

TATATAATTAATATTTTATTAAATAATTAAAATTAATATAAATTTTTATAAAATTTTTATACAATTAAATAATTTTAATAAGTAAATT

AATTTATAAATTATTAAGTTATATTAATAAATAAATTAGATAACTTAAATAAGTAAATGATTTTATAAAATTTATAAATTAAATTTTTTTTTTTTTTTTTTTTTTTTAATATATATATATATATATATAGATAATCTATATGTCTAAATATTAATATTTTT
ATAAATACATTAATATTATTTCAAAAAAAACCCTAATTTATATAAATAATGATTTAAATATAAATTAAAATCTATATTATTAAAGATAATATAATTATAGAAGGTTTTTACTTAATTATACAAAGCAATATTTAGTTAAATATTATATCCTTATGTTTA
ATAATTGTATATTTAATAAATATATATATATATATAATGTATATTTATTTTAATATAAATAATCGAGTTACATATATTTATTAAAATATTATTTGATAAATATTTAATGTAAATTTTAAATTACATTTAAAAATATATTCTTGAATATAGTTTTTAAATGT
AATTTAAAATTTATTAAATATTTATAGTTAAACATATAAATGTTTAATGGATAAACTTTTATTTATATACAACGCACTCATACTAAATTAAATTATTTAATTTGTATGTCAGTAATAATTTAGTTTTATAAAAAAATATTATTTTTTTCCTAAAAATAA
TAAATTATATGATAAAAGATATTTTTTTCATAAAAT

B

```
S_rueppellii_CR     AAATAATTTAATCATTTATTATATAAAAATAATAAATAATTAATATATTTTTAAATAATATTAATTAGTAAATATAATTTTATAACTTATATTTTATTAAATAATTAAAATTAAGTAATA
S_taeniata_CR       AAATAATTTAATAATTTATAATATAAAAATAATAAATTATTAATATATTTTTAAATAATATTAATTAGTAATAAAAATTTTATAATTTATATTTTATTAAATAATTGATATTAAGTAATA
S_philanthus_CR     AAATAATTTAATAATTTATAATATAAAAATAATAAATTATTAATATATTTTTAAATAATATTAATTAGTAATAAAAATTTTATAAATCATATTATATTAAATAATTATAATTAAGTAATA
                    ************ ****** ***************** **********************************  * ********** * ***** ************   ***********

S_rueppellii_CR     TCTACCAGAAAAA---AACTA-TTTTTTAATTAATATTTTATTTTATATTT-AATATAATATTTATCCCCTAAATAAATAATATTTCTAATATAAAATTTTAAAAGATATTTAATAATAA
S_taeniata_CR       GCTCCCGAAAAAAATCAACTA-TTTTTTTATTAAAATTCTATTTTATATTTAAATATATTATTTATCCCCTAAATAAATAATATTTCTAATTTAAAA-TTTAAAGAATATATAATAATAA
S_philanthus_CR     RCTCCCGAAAAAAATCAACTATTTTTTTTTATTAAAATTATATTTTATATTTAAATATATTATTTATCCCCTAAATAAATAATATTTCTAAATTAAAA-TTTAAATAATTTATAATAATAA
                     ** **  *****    ***** ****** ***** *** ************ ****** ******************************  ***** ******  ** * *********

S_rueppellii_CR     T---AGAAAAAAACTCAGTTTTATTTTTAAATTATAAAATTTAAAATTTTTGTTAATTTAGTTTTATAACTTATATTTTATTAAATAATTAAAATTAAGTAATATCTACCAGAAAA-AAAC
S_taeniata_CR       TTGAAAAAAAAACTCAGTTTTATTTTTAAATTTAAAAATTTTAAATTTTAATAAATTTAA-TATATAACTCATATTTTATTAAATAATTAAAATTAAGTACTAATTCCAAAAAAAATAATC
S_philanthus_CR     TTGAAAAAAAAACTCAGTTTTATTTTTAAATTTAWAAATTTTAAATTTTAATAAATTTAA------------------------------------------------------------
                    *   * **************************   ****** *******   * ******

S_rueppellii_CR     TATTTTTTAATTAATATTTTATTTTATATTTA--------------ATATA-ATATTTATCCCCTAAATAAATAATATTTCTAATATAAAATTTTAAAAGATATTTAATAATAATAGAAA
S_taeniata_CR       TATTTTTTCATTAAAATTTTATTTTATATTTAAATACATTATATATATATATATATATATATATACTAAATAAATATTATTTATAAATTAAAATTTA-------------------------
S_philanthus_CR     ------------------------------------------------------------------------------------------------------------------------

S_rueppellii_CR     AAACTCAGTTTTATTTTTAAATTATAAAATTTAAAATTTTTGTTAATTTAGTTTTATAACTTATATTTTATTAAATAATTAAAATTAAGTAATATCTACCAGAAAAA---AACTA-TTTT
S_taeniata_CR       ---------------------------------------------------ATTTATAATTTATATTTTATTAAATAATTGATATTAAGTAATAGCTCCCGAAAAAAATCAACTATTTTT
S_philanthus_CR     ------------------------------------------------------------------------------------------------------------------------

S_rueppellii_CR     TTAATTAATATTTTATTTTATATTT-AATATAATATTTATCCCCTAAATAAATAATATTTCTAATATAAAATTTTAAAAGATATTTAATAATAAT--AGAAAAAACTCAGTTTTATTTTT
S_taeniata_CR       TTTATTAAAATTCTATTTTATATTTAAATATATTATTTATCCCCTAAATAAATAATATTTCTAATTTAAAA-TTTAAAGAATATATAATAATAATTGAAAAAAAACTCAGTTTTATTTTT
S_philanthus_CR     ------------------------------------------------------------------------------------------------------------------------

S_rueppellii_CR     AAATTATAAAATTTAAAATTTTTGTTAATTTAGTTTTATAACTTATATTTTATTAAATAA TTAAAATTAAGTAATATCTACCAGAAAAAAACTATTTTTTAATTAATATTTTATTTTATA
S_taeniata_CR       AAATTTAAAAATTTTAAATTTTAATAAATTTAA---------------------------------------------------------------------------------------
S_philanthus_CR     ------------------------------------------------------------------------------------------------------------------------

S_rueppellii_CR     TTTAATATAATATTTATCCCCTAAATAAATAATATTTCTAATATAAAATTTTAAAAGATATTTAATAATAATAGAAAAAACTCAGTTTTATTTTTAAATTATAAAATTTAAAATTTTTGT
S_taeniata_CR       ----------------------------------------------------------------TATATAACTCATATTTTATTAAATAATTAAAATTAATATAAATTTTTATAAAATTT
S_philanthus_CR     ----------------------------------------------------------------TATATAATTAATATTTTATTAAATAATTAAAATTAATATAAATTTTTATAAAATTT
                                                                                       ****    * *          * ***    *        ******   *      * *

S_rueppellii_CR     TAATTTAGTTTTATAACTTATATTTTATTAAATAATTAAAATTTATAAAATTATATAAATAAATTTATTAGATAATTTAAATAAGTAAATAATTTTATAAATTTTAATGATGATTTTTTT
S_taeniata_CR       TTATACAATTAAATAATTTTAATAAGTAAATTTAAATATAAATTATTAAATTATATTAATAAATAAATTAGATAACTTAAATAAGTAAATGATTTTATAAAATTTATAAATTAAATTTTT
S_philanthus_CR     TTATACAATTAAATAATTTTAATAAGTAAATTAATTTATAAATTATTAAGTTATATTAATAAATAAATTAGATAACTTAAATAAGTAAATGATTTTATAAAATTTATAAATTAAATTTTT
                    * *   * **  **** **  **       *    *   ** ** **** ** ****** *******  ********* ************** ********** ****    ** *  *****
```

```
S_rueppellii_CR     TTTTTTTTTTTTTTTTTTTATATATATATATATATATATATAGATAATCTATATGTCTAAATATTAATATTTTTATAAATACATTAATATTATTTCAAAAAAAACCCTAATTTATATAAATG
S_taeniata_CR       TTTTTTTTTTTTTTTTTTTA-ATATATATATATATATATAGATAATCTATATGTCTAAATATTAATATTTTTATAAATACATTAATATTATTTCAAAAAAAACCCTAATTTATATAAATA
S_philanthus_CR     TTTTTTTTTTTTTTTTTTTA-ATATATATATATATATATAGATAATCTATATGTCTAAATATTAATATTTTTATAAATACATTAATATTATTTCAAAAAAAACCCTAATTTATATAAATA
                    ******************** ************************************************************************************************** 

S_rueppellii_CR     ATGATTTAAATATAAATTAAAATCTATATTATTAAAAATAATATAATTAATGAAGGGTTTTACTTAATTATAGAAAGCAAAATTTAATTAAATATTATATATTTATGTTTAGTAATCATA
S_taeniata_CR       ATGATTTAAATATAAATTAAAATCTATATTATTAAAGATAATCTAATTATAGAAAGTTTTTACTTAATTATGGAAAGCAATATTTAATTAAATATTATATCCTTATGTTTAATAATTGTA
S_philanthus_CR     ATGATTTAAATATAAATTAAAATCTATATTATTAAAGATAATATAATTATAGAAGGTTTTTACTTAATTATACAAAGCAATATTTAGTTAAATATTATATCCTTATGTTTAATAATTGTA
                    ************************************ ***** ******  *** * **************  ******* ***** *************  ********* ****  **

S_rueppellii_CR     TATTTAATAA--ATATATATATATATAATGTATATTTATTATAATATAAATAATCGAGTTATATATATTATTTAAAATTTTATTTGATAAATATTTAATCTAAATTTTAAATTACATTTA
S_taeniata_CR       TATTTAATAAATATATATATATATATAATAAATATTTATTTTAATATAAATAATCGAGTTATATATATTTATTAAAATATTATTTGATAAATATTTAATGTAAATTTTAAATTACATTTA
S_philanthus_CR     TATTTAATAA--ATATATATATATATAATGTATATTTATTTTAATATAAATAATCGAGTTACATATATTTATTAAAATATTATTTGATAAATATTTAATGTAAATTTTAAATTACATTTA
                    **********  *****************  ********* ******************** *******  ******* ******************** ********************

S_rueppellii_CR     AAAATATATTCTTGAATATAGTTTTTAAATGTAATTTAAAATTTATTAAATATTTATAGTTAAACATATAAATGTTTAATGGATACATTTTTATTTATATACAATACATTCGTACTAAAT
S_taeniata_CR       AAAATATATTCTTGAATATAGTTTTTAAATGTAATTTAAAATTTATTAAATATTTATAGTTAAACATATAAATGTTTAATGGATAAGTTTTTATTTATACACAACGCACTCATACTAAAT
S_philanthus_CR     AAAATATATTCTTGAATATAGTTTTTAAATGTAATTTAAAATTTATTAAATATTTATAGTTAAACATATAAATGTTTAATGGATAAACTTTTATTTATATACAACGCACTCATACTAAAT
                    *************************************************************************************   *********** ****  ** ** ********

S_rueppellii_CR     TAAATTATTTAATTTATATACCAATAATAATTTGATTTTACAAAAAAA-AAAATTATTTTCCTAAAAATAATGAATTATTTAATATAATATATTTTTTTCATAAAAT
S_taeniata_CR       TAAATTATTTAATTTATATGTCAGTAATAATTTAGTTTTATAAAAAAATATTATTTTTTTCCTAAAAATAATAAATTATATGATAAAAGATATTTTTTTCATAAAAT
S_philanthus_CR     TAAATTATTTAATTTGTATGTCAGTAATAATTTAGTTTTATAAAAAAATATTATTTTTTTCCTAAAAATAATAAATTATATGATAAAAGATATTTTTTTCATAAAAT
                    *************** ***  ** *********  ***** ******* *  *** **************** ****** * *** ** ******************
```

C

```
                                 10        20        30        40        50        60        70        80        90       100
                        ....|....|....|....|....|....|....|....|....|....|....|....|....|....|....|....|....|....|....|....|
S_rueppellii_repeat     TTTTATAACTTATATTTTATTAAATAATTAAAATTAAGTAATATCTACCAGAAAAAAACTATTTTTTAATT----AATATTTTATTTTAT-ATTTAATAT
S_taeniata_repeat-1     ........T....................G.T...........G..C..GA......T.A.C.A...TT..AT-T..A...C........AT..A.....
S_taeniata_repeat-2     A.......T....................G.T...........G..C..GA......T.A.C.A...TT..TATT..A...C........AT..A.....
S_philanthus_repeat     ........A.C.....A.............T............R..C..GA......T.A.C.A...TT..TATT..A...A........AT..A.....

                                110       120       130       140       150       160       170       180       190       200
                        ....|....|....|....|....|....|....|....|....|....|....|....|....|....|....|....|....|....|....|....|
S_rueppellii_repeat     AATATTTATCCCCTAAATAAATAATATTTCTAATATAAAATTTTAAAAGATATTTAAT---AATAATAGAAAAAACTCAGTTTTATTTTTAAATTATAAA
S_taeniata_repeat-1     .T................................T........A..G.-....A....AAT...TGA.A..........................TA...
S_taeniata_repeat-2     .T................................T........A..G.-....A....AAT...TGA.-..........................TA...
S_philanthus_repeat     .T...............................AT........A..T.-..T.A....AAT...TGA.A..........................TAW..

                                210       220
                        ....|....|....|....|...
S_rueppellii_repeat     ATTTAAAATTTTTGTTAATTTAG
S_taeniata_repeat-1     ....T.......AA.A......A
S_taeniata_repeat-2     ....T.......AA.A......A
S_philanthus_repeat     ....T.......AA.A......A
```

Percent Identity Matrix - created by Clustal2.1

1:	S_rueppellii_repeat	100.00	84.58	84.51	82.71
2:	S_taeniata_repeat-1	84.58	100.00	98.64	93.67
3:	S_taeniata_repeat-2	84.51	98.64	100.00	94.12
4:	S_philanthus_repeat	82.71	93.67	94.12	100.00
